# Sensor-Based Measurement Method to Support the Assessment of Robot-Assisted Radiofrequency Ablation

**DOI:** 10.3390/s24051699

**Published:** 2024-03-06

**Authors:** Hilda Zsanett Marton, Pálma Emese Inczeffy, Zsuzsanna Kis, Attila Kardos, Tamás Haidegger

**Affiliations:** 1 Gottsegen National Cardiovascular Center, 1096 Budapest, Hungary; martonhi@gmail.com (H.Z.M.);; 2 Faculty of Medicine, Semmelweis University, 1085 Budapest, Hungary; 3 Faculty of Mechanical Engineering, Budapest University of Technology and Economics, 1111 Budapest, Hungary; 4 Austrian Center for Medical Innovation and Technology (ACMIT), 2700 Wiener Neustadt, Austria; 5 University Research and Innovation Center (EKIK), Óbuda University, 1034 Budapest, Hungary

**Keywords:** atrial fibrillation treatment, radiofrequency ablation, sensor-driven intervention, robot-assisted ablation

## Abstract

Digital surgery technologies, such as interventional robotics and sensor systems, not only improve patient care but also aid in the development and optimization of traditional invasive treatments and methods. Atrial Fibrillation (AF) is the most common cardiac arrhythmia with critical clinical relevance today. Delayed intervention can lead to heart failure, stroke, or sudden cardiac death. Although many advances have been made in the field of radiofrequency (RF) catheter ablation (CA), it can be further developed by incorporating sensor technology to improve its efficacy and safety. Automation can be utilized to shorten the duration of RF ablation, provided that the interactions between the tissue and the RF tools are well understood and adequately modeled. Further research is needed to develop the optimal catheter design. This paper describes the systematic methodology developed to support robot-assisted RF CA characterization measurements. The article describes the custom instruments developed for the experiments, particularly the contact force limiter, the measurement procedure, and the evaluation of the results, as enablers for new results. The aim was to establish an objective, repeatable, robust measurement method and adjacent procedure.

## 1. Introduction

Atrial Fibrillation (AF) is the most common arrhythmia, which significantly increases the incidence of stroke and the risk of cardiovascular death [1,2]. Early detection and treatment of AF is crucial to improve clinical outcome. Radiofrequency (RF) catheter ablation (CA) is a standard method used for AF treatment [3]. During this procedure, the pulmonary veins are electrically isolated from the rest of the Left Atrium (LA). Robotic control and technology assistance, including medical 3D printing, can improve the accuracy of the procedures, as shown in previous studies [4,5]. Technology-driven methods, including robotics and Artificial Intelligence (AI), are gaining more importance in the medical field, supporting various aspects of digital surgery [6,7].

In this study, we developed a sensorized measurement platform that can be used as a generic setup for a range of RF ablation-related experiments. We used robot-assisted examinations to observe changes in temperature, time, and the impact of delivered energy on the size of RF ablation-related lesions. Our aim was to create a generic setup to present the concept of the experimental equipment, its functionalities, and the results of the initial testing.

### 1.1. Clinical Background

During normal cardiac sinus rhythm, the electrical activity of the heart is determined by the impulses generated by the sinus node [8]. In the case of AF, the normal sinus rhythm is disrupted by irregular signals, often originating from the pulmonary veins. This irregular atrial activation affects the efficiency of heart’s contraction and blood pumping and therefore the quality of life [9]. The reduction in blood flow can lead to blood clots in the left atrium, which significantly increase the incidence of stroke and cardiovascular death related to AF [10].

### 1.2. Radiofrequency Ablation Treatment of Atrial Fibrillation

Besides medical treatment, CA therapy can be used as a rhythm control strategy for AF. During the CA procedure, RF energy is used to create permanent tissue necrosis around the catheter, called RF lesion [11].

The procedure starts with inserting the catheter through the femoral vein to reach the LA after transseptal puncture. Three-dimensional (3D) electroanatomic mapping systems are typically used to provide real-time information on the atrium and facilitate better positioning of the ablation catheters. Once the ablation catheter is inserted into the LA and the tip of the catheter touches the heart muscle, the RF energy is directed to the tissue. Due to the increased resistance, the RF energy is converted into thermal energy, according to Joule’s law [12,13]. Proper tool–tissue contact is essential for successful energy delivery [14].

For a successful operation, transmural lesions (scar tissue that completely penetrates the heart tissue) are essential. During the current standard of care, small lesions are created by RF CA from one point to another at the predesignated areas; the technique is called point-by-point ablation. The maximum strength of the electric field is at the tip of the catheter; thereby, the maximum thermal energy is generated there. The delivered energy is closely correlated to the resulting temperature of the surrounding tissue. The actual power that can be delivered also depends on the impedance between the catheter and the tissue, which varies between two patients and even within the same body. The thermal energy deposition is affected by perfusion heterogeneity in biological tissues/organs. There are regions of high blood perfusion, moderate perfusion, low perfusion and zero perfusion or necrotic zones [15]. Selecting the right value of the applied power level (as an RF CA device feature) is important for the right size of the lesion; increasing power also increases the depth of necrosis [16].

Modern catheters are irrigated (liquid-cooled) to prevent the overheating of the tip of the catheter and the tissue, enabling higher power to be used for more efficient CA lesions [12,13].

Nowadays, a power of 50 watts RF CA is often applied to reduce the duration of interventions by increasing the energy for faster ablation, while also aiming to improve accuracy and safety [17]. However, due to the thickness of the atrial wall, even a minor error during the operation can lead to serious complications. While maintaining a relatively high contact force is essential for energy delivery, it can also easily perforate the wall of the atrium; therefore, choosing the right contact force is crucial for a safe procedure.

There are numerous bioengineering research issues that remain open in this field. These include developing detailed models of the human tissue–tool interaction [18], the heat dissipation of different catheter shapes [19], and the effect of applied forces during ablation [20].

### 1.3. Robot-Assisted Ablation

Although RF ablation is performed by certified experts in electrophysiology, the accuracy of the procedure could be improved through the use of CPS and robotic control. According to the literature [21], catheter robots are already available on the market for assisting such procedures, yet the standard of care still remains a minimally invasive intervention under human guidance [22,23].

The consistency of the RF ablation can be improved by using robotic targeting and positioning of the catheter tip. However, there are still many unresolved research issues regarding the optimal approach for high-energy CA. To address this fundamental problem, a systematic approach was followed to standardize the ablation process supported by a robotic arm. The aim was to extend our measurement platform to a generic setup, engineered for a range of RF ablation-related experiments. Our purpose was to improve the performance metrics of RF ablations via optimized high-energy procedures. One of the main challenges was to create a surgical environment that could imitate the RF ablation point of view as realistically as possible so that we could achieve outcomes comparable to real-life scenarios.

## 2. Materials and Methods

At the Antal Bejczy Center for Intelligent Robotics, we performed robot-assisted examinations for observing how changes in the temperature, time and delivered energy impacted the size of the RF CA lesions. In our research, focusing on the optimization of RF ablation of the heart, we employed complex robotic and sensors technology to design and implement a measurement and test capability for the objective assessment of various RF ablation treatment variations. In our study, we used pig heart tissue; it is often used for cardiovascular testing because its size and anatomy are very similar to the human heart. The matching anatomy of the pig heart and the human heart enabled a successful heart transplant with a genetically modified heart in 2022 [24].

We used human consumption-grade porcine hearts during the ex vivo experiments. The pieces of tissue necessary for the RF CA were systematically cut from a whole heart, obtained from a traditional wholesale market within less than 24 h of slaughter. The test surfaces, where ablation was performed, were prepared for the measurements. The RF intervention was performed at a visible and easily accessible area. It was crucial that the surface following the cut was smooth to ensure accurate measurement and metrics acquired from the specimen after the experiments. The test surface was carefully cleaned, if it was necessary, to avoid damage. The heart pieces were stored in a suitable refrigerator in the laboratory until the ablation. The porcine hearts were positioned on a copper table used for grounding. During the experiments, we aimed to study how the shape, the depth and the volume of the RF CA lesions change as a function of certain clinical parameters.

### Experimental Setup

**Figure 1:**
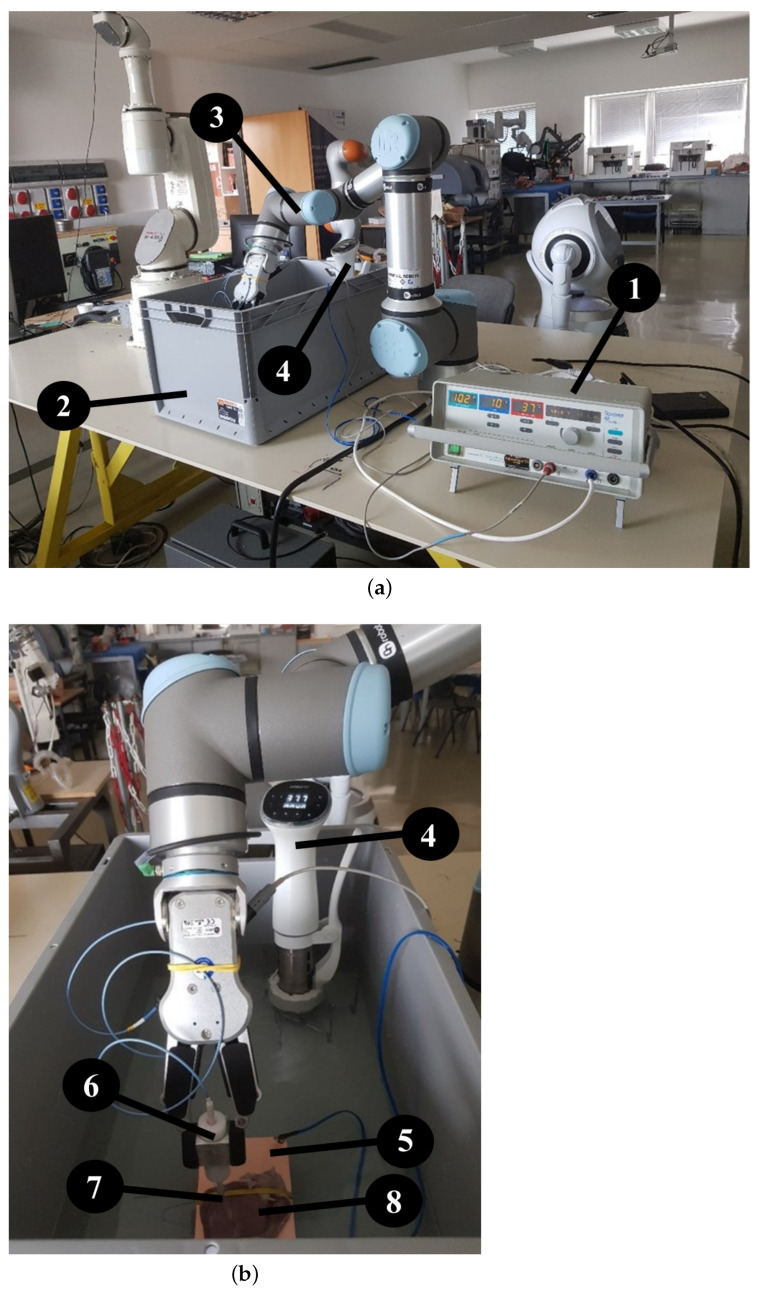
(**a**) The measurement setup for ex vivo porcine tissue RF ablation, set up at the Antal Bejczy Center for Intelligent Robotics, Óbuda University. 1: Stockert generator, 2: container, 3: Universal Robots UR16e robot arm and OnRobot RG6 gripper, 4: Ambiano Sous Vide machine. (**b**) The measurement setup for ex vivo porcine tissue RF ablation, set up at the Antal Bejczy Center for Intelligent Robotics. 4: Ambiano Sous Vide machine, 5: copper table used for grounding, 6: custom developed contact force limiter, 7: RF electrode, 8: porcine specimen.

To conduct our measurements, we employed an UR16e robot arm (Universal Robots, Odense, Denmark ) with an OnRobot RG6 gripper to prevent any inaccuracies that could have arisen at human operation. To carry out the ablation, we used a Standard Biosense Webster (Johnson & Johnson, New Brunswick, NJ, USA) Thermocool Catheter and a Stockert generator, on which we could adjust the power and the temperature. The experiments were conducted in a standard-sized container filled with physiological saline, which was used to replace blood since both liquids’ resistance is about 100 Ohm. The temperature of the saline was kept constant at an average internal body temperature of 37.5 °C, which was maintained by an Ambiano (Frankfurt, Germany) Sous Vide machine. Throughout the experiment, the temperature remained stable (Figure 1). We also took care to secure the stable position of the samples to ensure that the robot arm could not move the sample during movement and ablation. This was achieved with elastic bands that held the tissue specimen in place during the experiment without damaging the fabric as shown in Figure 1a,b.

## 3. Experiments and Outcomes

### 3.1. Contact Force Limiter Development

While most collaborative robotic arms are capable of setting and measuring the contact force at their Tool center point, their sensitivity remains relatively low, typically in the order of magnitude of 1 N. In contrast, the used model had a precision to 5 N, while during RF CA, usually 0.3 N is applied. To address this issue, an additional force limiter was included [20,25] with a spring contact force limiter developed and stabilized by the gripper.

**Figure 2:**
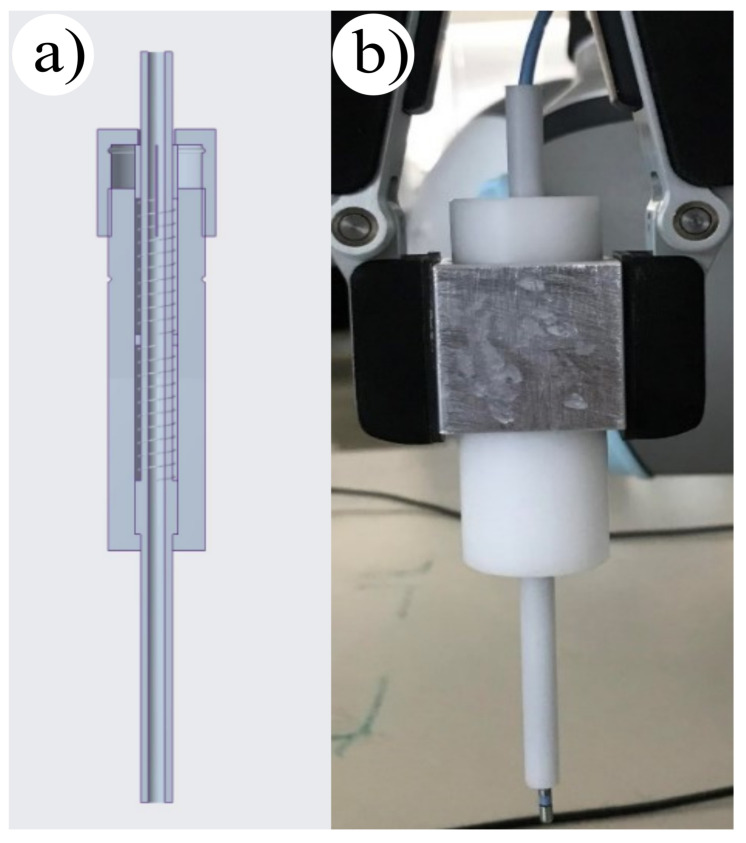
(**a**) CAD model of the spring contact force limiter, using two springs in series. (**b**) The fabricated add-on, able to set the contact force to a stable 0.3 N.

The mechanical add-on was designed based on Hooke’s law, which defines the correlation between the applied force and the deformation of the standard spring. The CAD model of the mechanical add-on is presented in Figure 2a. For this purpose a spring contact force limiter was applied, which we stabilized with the gripper.

Selecting the appropriate spring stiffness was deemed crucial, as small spring displacement can result in measurement inaccuracies, and a large indentation has structural limitations. To overcome this, two springs with s=0.13 N/mm individual stiffness were used in series, resulting in a shared stiffness of s=0.06 N/mm. The electrode was attached to the top of the limiter and passed through the tube part of the tool, where the required displacement for 0.3 N was marked. During the experiments, the same contact force could be set at every ablation point, which helped in reducing measurement inaccuracies (Figure 2). This method was proven to be effective in increasing the precision of collaborative robotic arms during RF CA.

### 3.2. Measurement Process

Our measurement setup allowed for ablation both in the atrium and the ventricle. However, due to the thickness of the atrial wall, we could make our measurements easier in the left ventricle. Ablation was performed at temperatures of 60, 65 and 70 °C for 10, 20 and 30 s, respectively, providing the possibility to ablate in both power control and temperature control modes.

With each setting combination, a series of five lesions was made. On each specimen, the same setting combinations were used instead of performing the complete five-lesion series on only one piece. In the experiments, point-by-point ablation was used, where lesions were made in row patterns, assisted by the UR robot, contributing to faster data processing. Having the proper distance between the points was very important because not only the primary but also the hardly visible secondary lesions were created during ablation. The formulating lesions do not cross each other because in the case of overlap, the exact dimensions cannot be determined.

**Figure 3:**
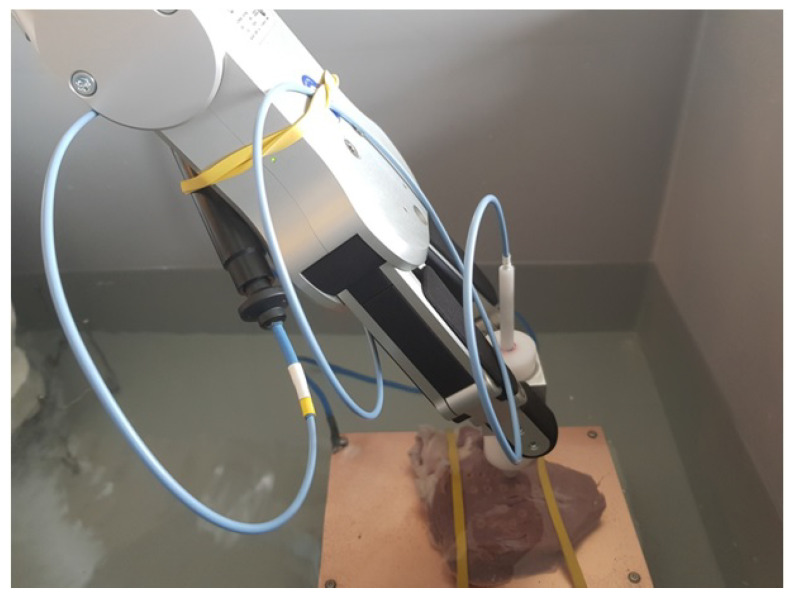
A close-up of the experiment, physiological saline solution in the tank and the ex vivo porcine heart on the copper table, during the process of RF ablation.

**Figure 4:**
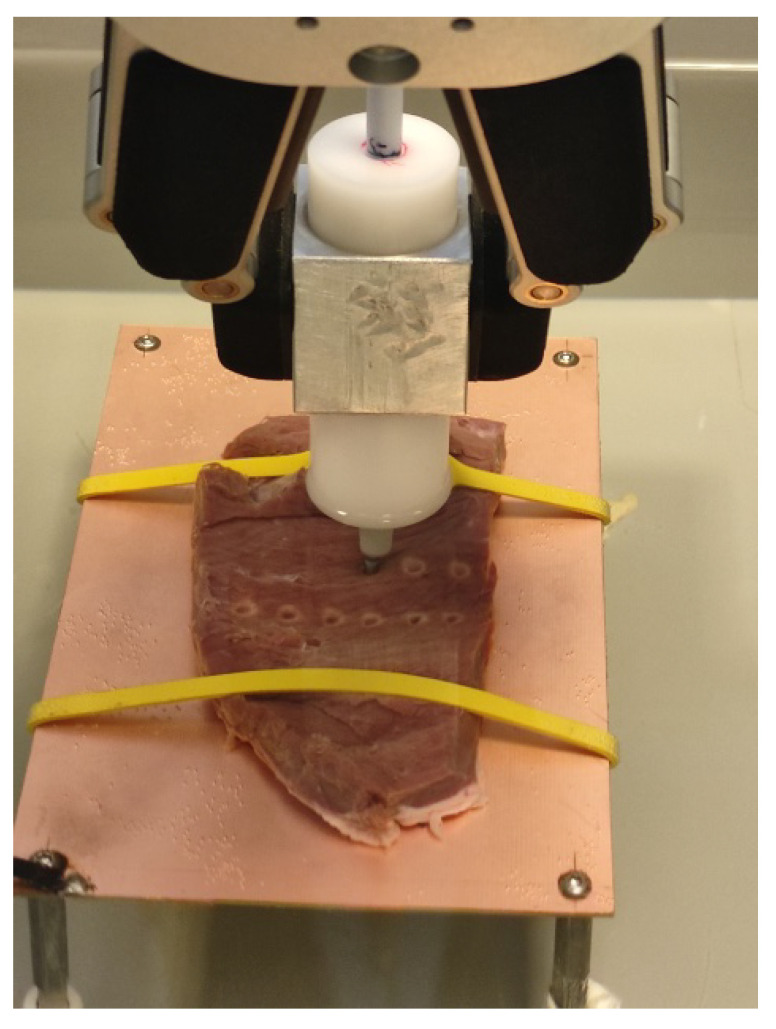
The ablation process supported by the sensorized, robot-assisted equipment.

For our measurements, we used the UR16e arm with the RG6 gripper to maintain the consistency of RF CA positioning, orientation and duration (Figure 3 and Figure 4). The robotic arm was a computer program controlled to reach each individual position, although it could also be switched to manual control if needed. To maintain a stable contact force, the displacement required for 0.3 N could be set with the custom force limiter, using the contact force adjusting device on the RG6 gripper. The Thermocool catheter was inserted into the inner tube of the contact force limiter. After reaching the aimed force, we initiated the ablation via the generator. Prior to ablation, the test surface was checked to ensure that it was free of large unevenness and suitable for ablation. After completing a series of ablations, we made top and cross-section cuts and scanned the porcine heart segments with the lesions. We used Inkscape software (http://gitlab.com/inkscape/inkscape, accessed on 1 January 2020), to read the dimensions and fed the information into a Microsoft Excel table for assessment.

## 4. Discussion

AF is a cardiac arrhythmia that affects an increasing number of patients globally. This has led to the development of technological innovations in the field of AF ablation therapy. In the near future, robotics will offer an opportunity to shorten not only the CA procedure time but also the recovery period. Robots are already being used in various clinical settings to improve the accuracy and outcome of surgical procedures, with an increasing level of autonomy [26] in these systems. Apart from the numerous cardiac catheter robots [27], there are also systems aiming for RF ablation [28].

The mid-term future of robot-assisted cardiac ablation particularly holds significant promise for advancing the treatment of cardiac arrhythmia. Patients and caregivers may benefit from the following:

- Improved Precision: Future robotic systems will incorporate enhanced sensing technologies and algorithms for better navigation and targeting within the heart chambers. This would enable more precise ablation of the arrhythmogenic tissue while minimizing damage to surrounding healthy tissue [29].
- Miniaturization: As robotics and microtechnology continue to advance, we may see the development of smaller robotic platforms capable of navigating through intricate cardiac structures with greater ease. Miniaturization could also facilitate access to regions of the heart that are currently challenging to reach [30].
- Integration of Imaging Modalities: Integration of advanced imaging modalities such as Magnetic Resonance Imaging (MRI) or 3D cardiac mapping into robotic systems can provide real-time visualization of the cardiac anatomy and electrical activity [31]. This integration would enhance procedural planning and execution, leading to better outcomes for patients.
- Teleoperation and Remote Assistance: Robotic systems may incorporate teleoperation capabilities, allowing expert electrophysiologists to perform procedures remotely. This could improve access to specialized care in underserved areas and enable collaboration between physicians across geographical distances, or even supporting astronauts in space [32].
- Artificial Intelligence and Machine Learning: Integration of AI and ML algorithms can aid in real-time decision-making during procedures. These algorithms can analyze physiological data and provide insights to assist physicians in identifying optimal ablation targets and predicting treatment outcomes [33].
- Autonomous Navigation: Advancements in autonomous navigation technologies may enable robotic systems to navigate the cardiac anatomy independently, guided by preoperative imaging and AI algorithms [34]. This autonomy could potentially reduce procedural times and enhance consistency in treatment delivery.
- Enhanced Safety Features: Future robotic platforms are likely to incorporate advanced safety features such as collision avoidance mechanisms and real-time monitoring of vital parameters. These features would further mitigate procedural risks and ensure patient safety during robotic-assisted ablation procedures. Cyber–Physical Systems (CPSs) can not only facilitate the realization of novel surgical treatment methods but also support the development of improved regulatory compliance [35].
- Cost Reduction and Accessibility: As technology matures and becomes more widespread, the cost of robotic-assisted procedures may decrease, making them more accessible to a larger patient population. This could democratize access to advanced cardiac arrhythmia treatment options globally. Recent research shows the growing interest and Willingness To Pay (WTP) of patients regarding robot-assisted surgeries [36].
- Improved Sustainability Aspects: Recently, the sustainability and ESG (Environment, Social and Governance criteria) aspects of such technology-heavy surgical interventions have also come under the spotlight [37], along with systematic engineering methods for ethically aligned system development [38].

Overall, the future of robot-assisted cardiac ablation holds great promise for improving treatment outcomes, reducing procedural risks, and enhancing patient care in the field of electro-physiology. However, for now, the standard of care remains the manual guidance of the RF catheters, which requires new, validated techniques for faster and more consistent tissue ablation while guaranteeing safety.

Increasing the power of the RF CA leads to faster necrosis, but it also increases the risk for perforation with the larger contact forces. These critical parameters were objectively measured in a custom-developed experimental setup, which has provided valuable insight into the technique.

In conclusion, the use of robotics in AF ablation therapy is a promising area of research that has the potential to improve patient outcomes and reduce recovery time.

### 4.1. Strengths of the Experimental Study

**Figure 5:**
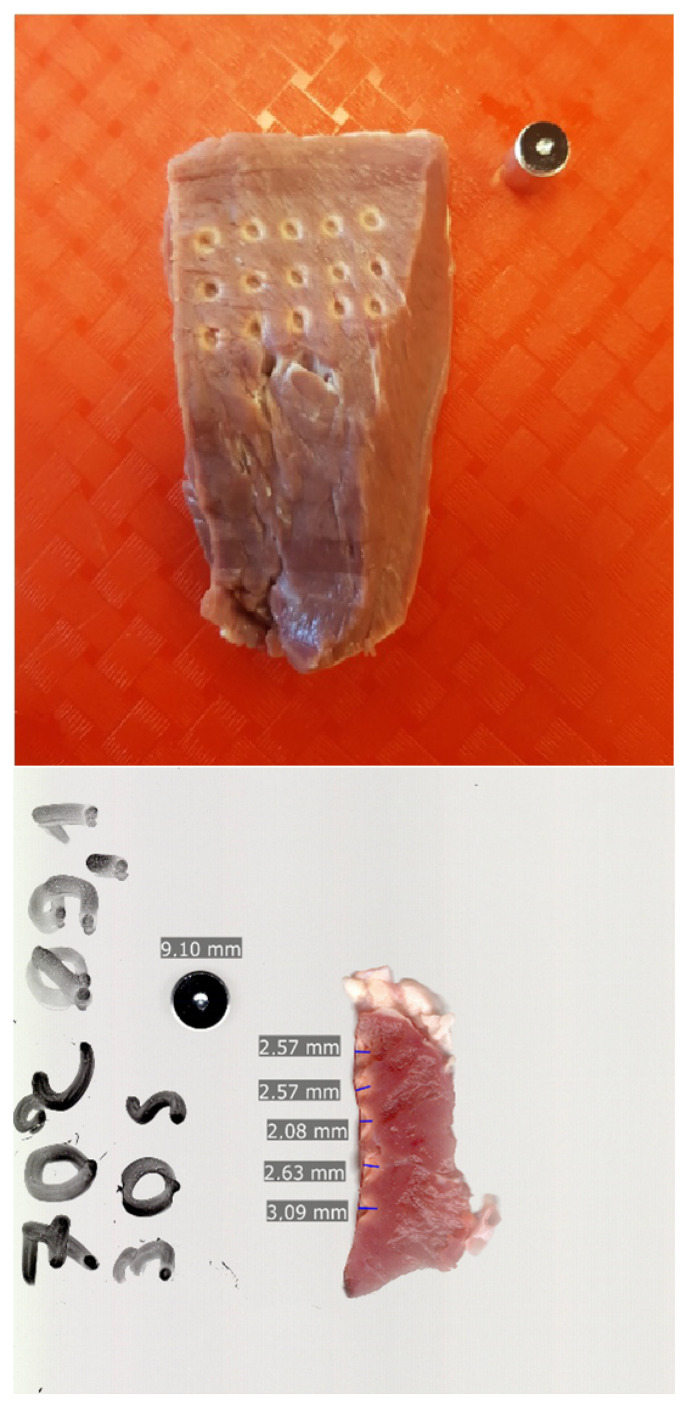
Top and cross-section scanned and scaled images of the porcine hearts with lesions.

The study environment we designed and built met our expectations. While it is not possible to replicate the exact operating room conditions during an ex vivo experiment, our measurement setup and methodology provided a reasonable compromise to achieve repeatable and objective assessment. The entire setup was easy and quick to assemble and disassemble. We have successfully created a reproducible experimental setup for measuring the lesions generated by the RF ablation of ex vivo porcine tissue (Figure 5).

Our original aim was to develop a device that could easily and quickly create RF ablation lesions and measure their critical parameters, including diameter and depth (see Section 4 Discussion) [39]. The measurement platform underwent rigorous testing for routine assessment of ex vivo porcine hearts, and then we intended to make a series of measurements based on the above.

While modern ablation systems can continuously measure the contact force, we were able to adjust the contact force only to a certain value during our experiments. However, with the contact force limiter, we were able to adjust the displacement to the required contact force at one point. It is essential to highlight that during movement, the force also varies due to the irregularities of the heart surface. Measuring the force instead of adjusting is necessary for improvements, so it is recommended to use a digital method instead of the analog one.

### 4.2. Limitations

The electrode used for our research had a diameter of 2.5 mm, but it was easily deflected due to its design. In a surgical environment, stability is provided using steerable sheaths, which were not available for our research. Another movement-related issue we faced was the stuttering motion of the electrode while dragging. Due to the fibrous tissue structure of the heart, the small diameter electrode repeatedly got stuck, preventing smooth movement and energy transfer. These last two problems could be solved with a device attached to the end of the electrode, which would replace the sheath. Moreover, the porcine hearts used in the research were not in vivo, and the tissue behaved differently than a living organ would. For accurate measurement results, it is recommended to use a heart that is as fresh as possible.

## 5. Conclusions and Future Research Aims

We have successfully demonstrated a systematic approach to assessing the critical parameters of radiofrequency cardiac ablation using a custom robot-assisted experimental setup. We employed an UR 16e robot with a RG6 gripper and a purpose-built force limiter to objectively evaluate the lesions produced on ex vivo porcine heart tissue. This technique enabled us to conduct a more precise and consistent assessment of the ablation process compared to traditional methods.

During our experiments, we mainly utilized a point-by-point ablation technique. Our objective was to conduct a series of measurements using different settings to evaluate lesions based on diverse technical parameters. In both power and temperature modes, we scrutinized the size, the depth, and the width of the lesions to determine which mode produces more effective lesions. During the assessment of the measurements, we aimed to explore correlations between various technical parameters to contribute valuable insights to the field of lesion science.

However, in the future, we plan to conduct a deeper investigation of the dragging ablation technique to utilize the force-controlled robots’ extensive capabilities. Our ultimate goal is to create a medical simulator that replicates the real-life surgical conditions for high-fidelity ex vivo research data. This device would be able to stimulate heart beating with pulsating movement, a crucial influencing factor that is not present in most ongoing laboratory research. By simulating realistic conditions, we aim to better understand how the ablation process affects the heart and produce more reliable and accurate results.

In the future, a heart motion-tracking ablation device would offer more predictable outcomes. Our further goal is to develop a system capable of following the heart’s anatomical motion to ensure constant contact force and position. However, before we can achieve this goal, we need to gain more information on the formation of lesion. By understanding how the lesion forms, we can create a more effective and precise ablation process.

Although there are still challenges to be overcome, the development of new techniques and the use of objective measurements will allow for greater precision and efficacy in this important medical field.

## Data Availability

Data are contained within the article.

## Abbreviations

The following abbreviations are used in this manuscript:

AF	Atrial Fibrillation
AI	Artificial Intelligence
CA	Catheter Ablation
ESG	Environment, Social and Governance (criteria)
LA	Left Atrium
ML	Machine Learning
MRI	Magnetic Resonance Imaging
RF	Radiofrequency
WTP	Willingness To Pay
